# The Smart Predicting of Algal Concentration for Safer Drinking Water Production with Sensor Data

**DOI:** 10.3390/s23115151

**Published:** 2023-05-28

**Authors:** Han Yu, Jing Li, Linda Holmer, Stephan J. Köhler

**Affiliations:** 1 Swerim AB, Process Metallurgy, SE-971 25 Luleå, Sweden; han.yu@swerim.se; 2 Division of Water Resources Engineering, LTH, Lund University, John Ericssons väg 1, SE-223 63 Lund, Sweden; 3 Görvälnverket, Norrvatten, Vattenverksvägen 20, SE-175 47 Järfälla, Sweden; linda.holmer@norrvatten.se (L.H.); stephan.kohler@norconsult.com (S.J.K.); 4 Norconsult, Bangårdsgatan 13, SE-753 20 Uppsala, Sweden

**Keywords:** algal bloom, lake, AI, drinking water supply, neural network, closed system

## Abstract

To better predict the timely variation of algal blooms and other vital factors for safer drinking water production, a new AI scanning–focusing process was investigated for improving the simulation and prediction of algae counts. With a feedforward neural network (FNN) as a base, nerve cell numbers in the hidden layer and the permutation and combination of factors, etc., were fully scanned to select the best models and highly correlated factors. All the factors involved in the modeling and selection included the date (year/month/day), sensor data (temperature, pH, conductivity, turbidity, UV254-dissolved organic matter, etc.), lab measurements (algae concentration) and calculated CO_2_ concentration. The new AI scanning–focusing process resulted in the best models with the most suitable key factors, which are named closed systems. In this case study, models with highest prediction performance are the (1) date–algae–temperature–pH (DATH) and (2) date–algae–temperature–CO_2_ (DATC) systems. After the model selection process, the best models from both DATH and DATC were used to compare the other two methods in the modeling simulation process: the simple traditional neural network method (SP), where only date and target factor as inputs, and a blind AI training process (BP), which considers all available factors as inputs. Validation results show that all methods except BP had comparable results for algae prediction and other water quality factors, such as temperature, pH and CO_2_, among which DATC displayed an obviously poorer performance through curve fitting with original CO_2_ data compared to that of SP. Therefore, DATH and SP were selected for the application test, where DATH outperformed SP due to the uncompromised performance after a long training period. Our AI scanning–focusing process and model selection showed the potential for improving water quality prediction by identifying the most suitable factors. This provides a new method to be considered in the enhancing of numerical prediction for the factors in water quality prediction and broader environment-related areas.

## 1. Introduction

Harmful algal blooms (HABs) have caused severe threats to water bodies and the supply of drinking water globally in recent decades [1,2,3]. Today, in Sweden and certain other regions across the globe, intensive algal blooms are mostly limited to lakes, which release legacy phosphorus from lake sediments. The blooming occurs during periods when oxygen depletion in lower parts of the water body trigger anoxic conditions in the sediment. Intensive blooms lead to several societal challenges. Neurotoxins and toxic phytoplankton as well as the reduction in oxygen concentration in water bodies [4] can be a direct risk to the health of ecology and humans. Algal blooms may also pose indirect problems for drinking water production, such as the obstruction of filters at the intake or even sand filters. The current removal methods for algae cells include coagulation [5] (Lin et al., 1971), flotation [6] ultrafiltration [7], activated carbon, etc. The removal of dissolved toxins caused by algae is an even greater challenge. Especially toxins with small molecular weights of below 400 daltons, such as Nodularins, would require nanofiltration for quantitative removal [8]. Cyanotoxin release mechanisms and the amount of toxins released are largely unknown. Proxies for other factors, such as studying the algae biovolume, species composition, influencing factors, and developing early indications of/predictions for the presence of algal blooms, are necessary [9]. Rapid prediction systems for algal blooms are urgently needed to guide necessary precautions and reduce the possible damage and loss for ecology and humans in advance.

The main factors causing algal blooms have been summarized in past research works. For instance, it is agreed that temperature; nutrient status [10,11], especially the N/P ratio; and light conditions are the main drivers of cyanobacterial blooms. The exact trigger mechanism of blooms, however, is still difficult to quantify. Often, they depend on local conditions [12]. Under those circumstances, AI-based algorithms with no need for physical/chemical equations may assist in early warning systems.

Recently, several studies highlighted the use of numerical methods based on AI that allow the prediction of water quality and, in some cases, the occurrence of algae [13,14,15]. Saboe et al. (2021) conducted a prediction of algae concentration and water quality parameters via a new combination of microbial potentiometric sensor signals and machine learning tools, resulting in considerable prediction performances [13]. Based on the simple application of AI in a water quality simulation, Ahmad et al. (2019) further conducted the denoising through a neuro-fuzzy inference system (WDT-ANFIS) during AI training, improving the prediction performance significantly [14]. For the selection of suitable AI models, Wang et al. (2022) compared traditional fitting with various AI methods for the prediction of flocculant dosage, suggesting the Elman neural network as the prior choice in this case [15]. Recent studies have covered valuable areas such as new combinations of sensors, experimental measurements, and AI models, highlighting the synergistic work between AIs and the selection of suitable AI algorithms, among other things.

However, research still neglects significant aspects which may influence the prediction performance. For instance, methods for selecting the best parameters, neuron numbers, and starting points have rarely been reported. The selection of input/output variables is either disregarded as an important step or conducted only using the simple statistics of correlations between two variables. This approach lacks the ability to identify the role of interdependent variables. Auto-deep learning (AutoDL) applications allow for autonomic data selection and the construction of layers in models, which can reduce human intervention in water quality prediction [16]. However, contrary to what might be expected, AutoDL did not perform better. As a result, high and stable performance in the AI prediction of multiple factors is still an active research field.

Therefore, in this work, we aimed to establish a new fully AI scanning–focusingprocess for selecting the best model to further address the above issues and improve the prediction performance of an early warning system for the occurrence of algae based on multiple water quality factors. The code is programmed to select the best prediction models with most suitable water quality factors as well as neuron numbers in the hidden layer, random factors during the training process, etc. We define the best prediction models with the best combination of suitable factors as so-called “closed systems”, in which all included factors are highly correlated with each other. These identified best models of closed systems are then compared with other methods. The considerable performance of the closed system approach demonstrates its potential to improve the prediction performance through new aspects. Water quality factors, including algae concentration (cell numbers), water temperature, pH, conductivity, turbidity, dissolved organic matter, calculated CO_2_ concentration and date (year/month/day) are involved as inputs and outputs. To be exact, in each training and application of the prediction model, the inputs are date and selected factors, while the outputs are the predicted values of these selected factors, except for the date.

This study aims to improve the prediction performance with the new full-scanning–focusing process for the best model selection with the most suitable combination of factors (closed system), rather than pursuing an extreme highly accurate prediction result during the calibration period.

In this paper, we discuss the threat of algae bloom and the gap in the knowledge of algae prediction and early warning systems and present our solution of a full-scanning–selecting system to improve the prediction performance of algae concentration (see Section 1). Section 2 includes the measurement and data collection processes as the basis of the model as well as the modeling and prediction study design. Section 3 mainly focuses on the comparison of the prediction performances of various models in training, validation, and application periods as well as the corresponding analysis and discussion. This is to demonstrate the performance and features of our new system. Finally, we summarize our study and discuss the limitations and future perspectives of this work.

## 2. Materials and Methods

**Figure 1:**
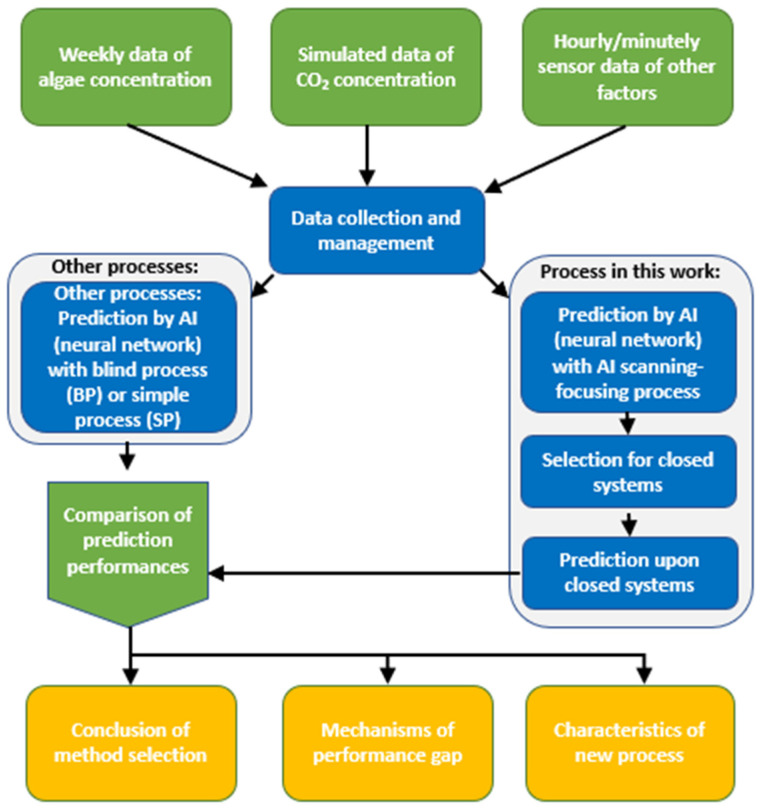
The flowchart of the methodology of this work.

In this work, we aimed to introduce a new scanning–focusing AI process for the prediction of algae concentration in lake water and several other factors crucial for water treatment. The flowchart of this work is shown in Figure 1. The raw data are based on weekly measured algae concentrations, simulated CO_2_ concentrations, and hourly/minutely measured values of other factors, such as temperature, pH value, conductivity, turbidity, and dissolved organic matter. Two AI prediction processes: (1) a blind AI training process with all factors considered (BP) as inputs and (2) a simple process (SP) with only the date and target factor as inputs considered (closed system). The prediction performances of various factors with different AI processes are compared and summarized. 

### 2.1. Description of the Görväln Drinking Water Treatment Plant

**Figure A1:**
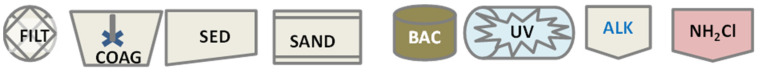
The water treatment consists of a microfilter prefiltration, coagulant dosing using Al_2_(SO_4_)_3_, sedimentation basin (SED), filtration through rapid sand filter (SAND), a passage through biologically active carbon filters (BAC), an UV treatment, pH adjustment (ALK), and disinfection with chloramine.

The Görväln drinking water plant (DWTP) is located on the eastern side of Sweden’s third largest lake, Lake Mälaren. The plant is run by municipal water company named Norrvatten and produces drinking water for around 600,000 people in the greater Stockholm area. The process is a classical coagulation–rapid sand filtration process (Figure A1).

The DWTP intake is located inside the Görväln basin, where two water sources meet, one high in alkalinity (>1.2 mM) and high in organic carbon (>10 mg L^−1^) flowing from the north, and the other low in alkalinity (<0.5 mM) and generally much lower in organic carbon (<7 mg L^−1^) flowing from the western part of the lake. The basin has a turnover time of around 3 months. Nutrient concentrations of P–PO_4_ usually vary between 5 to 50 μgL^−1^ while nitrate concentration lies between 300 to 1800 μgL^−1^. During spring and late autumn, chlorophyll concentrations in the basin may rise to 50 mg L^−1^ and the presence of blue–green algae has been repeatedly confirmed.

The DTWP is equipped with several online sensors that register several parameters (Table 1) at high temporal resolutions. These signals are used to control the dosing of the coagulation process and to observe important changes in water quality.

#### 2.1.1. Description of Algal Cell Count Method

Algae counts were carried out using an inverted microscope. For this purpose, 500 mL of water was sampled at the intake and 50 mL was treated with Lugol’s solution and then left to settle for three days for sedimentation using an utermohl chamber (Hydrobios).

The final algal counts are given as the number of cells per liter of water (cells L^−1^). On a few occasions data for both algal cell number and chlorophyll content are available. Based on variance in size, it is not necessarily expected that the cell numbers and chlorophyll will correlate. In our case, we found a significant relationship, which indicates that a measurement of 4 × 10^6^ cells corresponds to around 30–40 ug L^−1^ chlorophyll. This comparison allows us to convert the observed cell number to a hypothetical chlorophyll concentration in raw water. In general, in natural lake water for managing recreational waters, chlorophyll-a < 10 ug L^−1^ is regarded safe, chlorophyll-a >10 ug L−1 with a dominance of cyanobacteria would pose a relatively low probability of adverse health effects, chlorophyll-a >10 ug L^−1^ and <50 ug L^−1^ would pose moderate probability of adverse health effects and chlorophyll-a > 50 ug L^−1^ would pose high probability of adverse health effects [17].

#### 2.1.2. Calculation of Carbon Dioxide

The photosynthetic activity of algae leads to an uptake of carbon dioxide from the water body. While no direct measurements of carbon dioxide were available, it is possible to accurately calculate carbon dioxide concentration if pH, temperature, and alkalinity are known.

**Figure A2:**
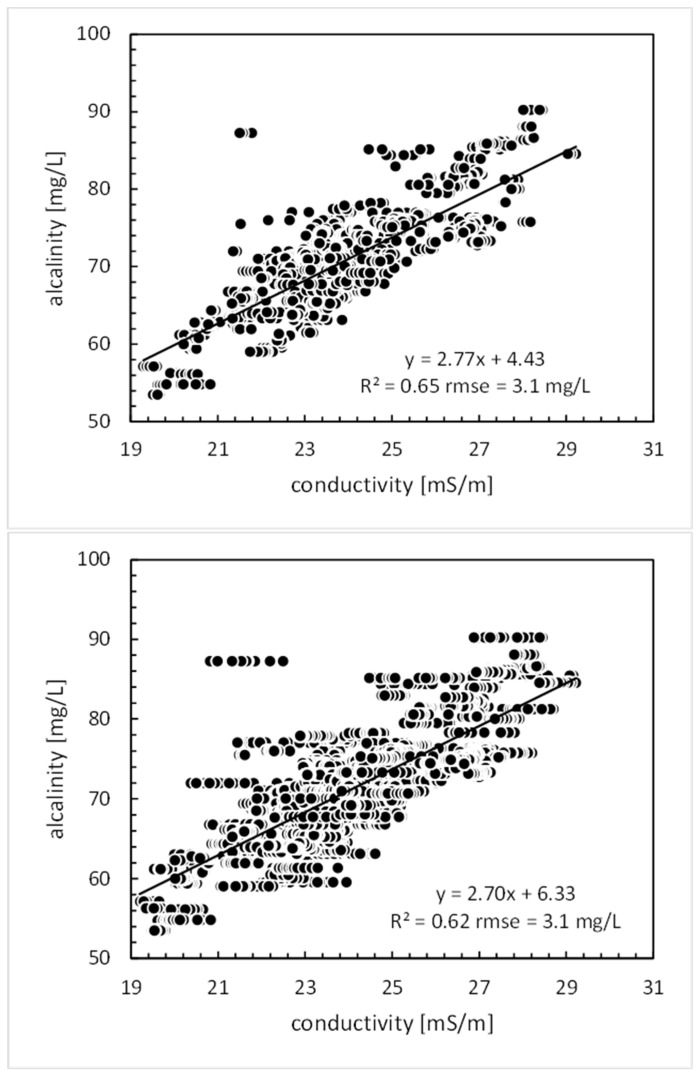
Example of observed correlation between conductivity (mS m^−1^) and alkalinity as HCO_3_ (mg L^−1^) both when using data within 6 h (**upper panel**) and 24 h (**lower panel**).

**Figure A3:**
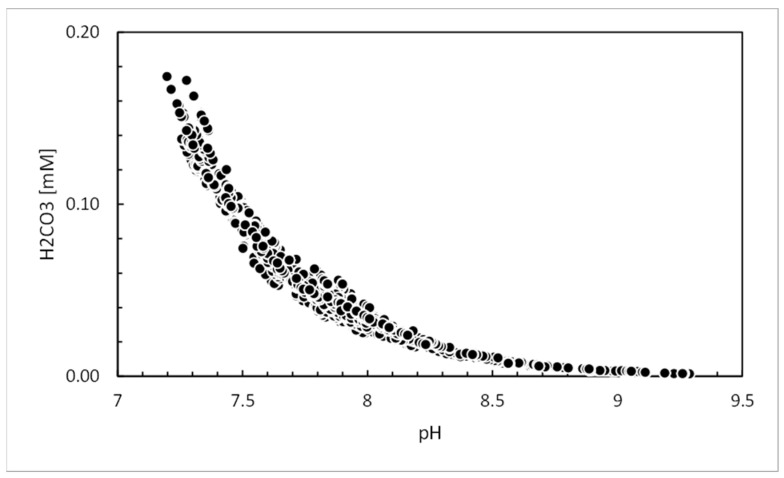
Calculated concentration of dissolved carbonic acid (H_2_CO_3_ (mM) as a function of pH and temperature covering the range between 0.5–12.5 °C and pH between 7.2–9.3.

Carbon dioxide concentrations were calculated based on estimates for alkalinity and measured pH and water temperature using the reactive transport model PHREEQC, which is freely available from the USGS site (version 2.17 for Microsoft Windows; USGS, 2020) [18,19]. In surface waters that are metastable with respect to calcite (i.e., no actual dissolution and precipitation occurs due to the low deviation from the calcite saturation index), pH is controlled by alkalinity, the presence of organic matter, temperature, and carbon dioxide only. The presence of bacteria may respire organic matter by increasing carbon dioxide concentration while the presence of photosynthetic algae leads to a decrease in carbon dioxide because of photosynthesis. While pH, conductivity, and temperature data were available at 5 min intervals, alkalinity was only measured weekly. In the high alkalinity lake water studied at this site, a strong correlation exists between conductivity and alkalinity. This observation can be used to produce a time series with much higher temporal resolution for calculated carbon dioxide concentration. An analysis revealed that the correlation between alkalinity and conductivity slightly worsens (Figure A2) if data are included that are more than 6 h away from the actual weekly measurements. Based on that criterion, we had access to around 2000 data points in the period 2015–2022 as data for the chemical equilibrium calculation. The correlation between the pH and content of dissolved carbon acid is displayed in the Appendix A (Figure A3).

### 2.2. Modeling for Prediction and Early Warning Systems

We introduce a new AI-focusing process to model the interaction among several vital water chemical factors in a waterbody for prediction and early warning systems. This aims to conduct high quality modeling in complex environments, search for a relatively closed system for prediction, improve the prediction performance of AI-based modeling, and guide the reduction in necessary monitoring.

Firstly, the interaction among these data of parameters in the water source was modeled through a feed-forward neural network (FNN) for prediction and analysis, carried out in Matlab. This is due to the strong feasibility of neural networks to simulate complex systems. In this case, factors with available data, such as date, algae concentration, water temperature, pH, conductivity, turbidity, and dissolved carbon dioxide concentration, from between January 2015 to June 2020, were involved in training and validation. The data from 2015 to 2019 were employed for training, while those from 2020 were used for validation. Data for 2022 was used for observation purposes. The fitting performance was tested via MSE and R^2^. Both were calculated from the normalized value of the simulated and original values of all involved variables to represent an integrated fitting performance.

In the FNN model, the construction of 1 input layer, 1 hidden layer, and 1 output layer were applied. During training, 1 to 10 neurons in hidden layers were fully scanned and 10 parallel training tests were conducted with the same training parameter settings to consider the influence of random factors during training. This is to avoid occasionality and to select the proper model construction for each condition. To study the possible advanced influence and delayed reflection between factors, a matric input with 2 dimensions for date and various parameters were applied. Here, we considered 7 weeks as the date range for the input in the training to project data one week into the future for the output.

Secondly, to improve the prediction performance by selecting a satisfactory closed system and to reveal relativity and interaction mechanics between factors, an AI scanning–focusing process was conducted. We analyzed the involvement of various factors in the model outcome. For the prediction of algal bloom, the date and algal concentration were the basic input factors in our modeling, with the consideration of the permutation and combination of the 6 other factors as extra inputs, resulting in a total of 63 combinations. The FNN modeling was processed for each of those sets and prediction performances were compared. Subsequently, relative closed systems that of better performance were selected, which also indicated factors of higher relativity. and Here, we applied the theory of Granger causality [20] for the judgment of closed systems and relativity. If the involvement of one factor can improve the fitting performance of the whole system, it is retained. Finally, the systems with the highest performances are selected. The proper construction of the FNN model in the corresponding conditions was achieved.

## 3. Results and Discussion

### 3.1. Model Selection and Performance

During the training and validation of a neural network model, all possible 63 combinations of factors were explored. The constructions with 1 to 10 neurons in the hidden layer were scanned, resulting in 630 conditions. The parallel training was replicated 10 times for each condition.

In the scanning process, the fixed factors of date and algae concentration with the six other flexible factors were tested in the model training, and the closed systems with the best corresponding high performance prediction model were selected and evaluated later in the validation stage. The output factors were the same as the inputs, except for the date.

As a result, for each combination, the highest prediction performances were found when the number of neurons in the hidden layer was the same as that of the output factors. In addition, the following two relative closed systems, date–algae–temperature–pH (DATH) and date–algae–temperature–CO_2_ (DATC) were found to have the highest average integrated prediction performances. All other combinations of variables were discarded. DATH and DATC resulted in the highest average R^2^ values (0.715 and 0.688, respectively) in the validation period with three neurons in the hidden layer. The factors therein represented higher relativity than other variable sets in this study.

**Figure 2:**
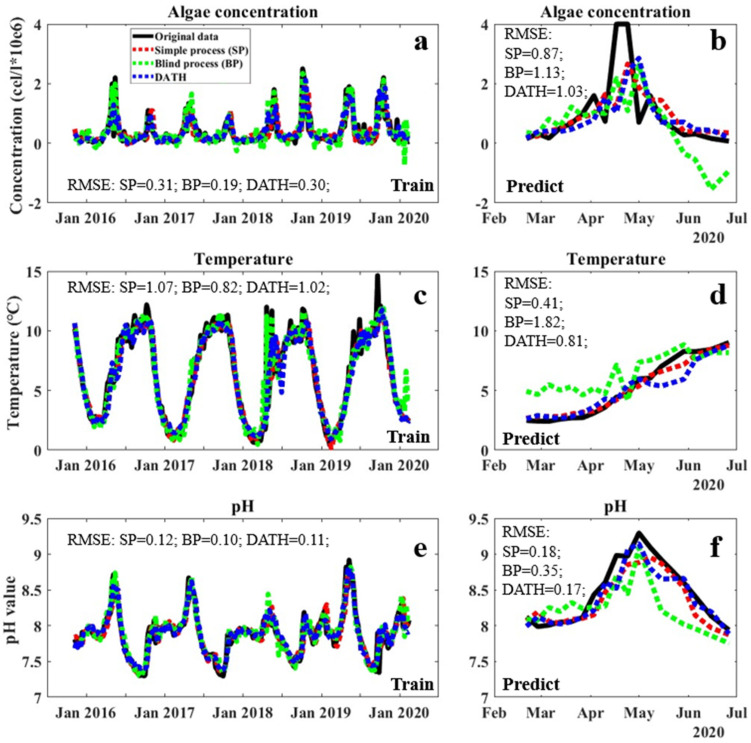
Fitting performances of algae concentration, temperature, and pH in the training period (**left**) and validation (**right**) period using the DATH closed system and other methods. BP refers to a blind AI training process with all factors considered as inputs, and SP refers to a simple process with only the date and target factors as inputs. Algae concentration model performance in the training period (**a**) and validation period (**b**); Temperature model performance in the training period (**c**,**d**); pH model performance in the training period (**e**) and validation period (**f**).

**Figure 3:**
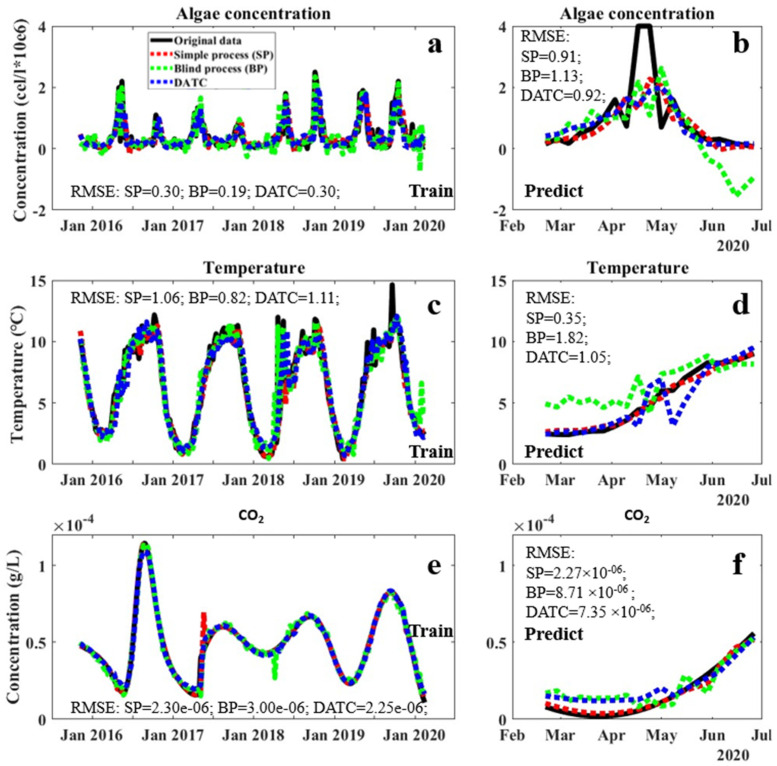
Fitting performances of algae concentration, temperature, and CO_2_ concentration in the training (**left**) and validation (**right**) periods using the DATC closed system and other methods. BP refers to a blind AI training process with all factors considered as inputs, and SP refers to a simple process with only the date and target factors as inputs. Algae concentration model performance in the training period (**a**) and validation period (**b**); Temperature model performance in the training period (**c**,**d**); pH model performance in the training period (**e**) and validation period (**f**).

The model with highest prediction performances from DATH and DATC were selected for the evaluation of fitting performances in the training and validation periods, as shown in Figure 2 and Figure 3, respectively, with the statistical evaluation of prediction performance shown in Table 2 and Table 3. The blind fittings with all factors involved (BP) and simple fittings with only date and the targeted factor included (SP) as the control. In general, both DATH and DATC models performed considerable fittings for all involved factors and avoided overfitting in the training and validation periods (Figure 2 and Figure 3). In the validation period, their performances were comparable or slightly lower to those of simply fitting (SP) in general, which is supposed to perform well in training and validation within the closed system, by ignoring interruptions from other factors. The performance gap between DATH/DATC and blind fitting was apparent in the validation period of algae concentration, temperature, and pH value (Figure 2b,d,f and Figure 3b,d,f). This effect could be due to disturbances from factors that are irrelevant for algae occurrence without the selection of a closed system. Between DATH and DATC, despite the similar R^2^ for algae prediction in training and validation periods, obvious mismatches occurred in original carbon dioxide concentrations in the prediction stage (Figure 3f shows the data for DATC (green mark)). This indicates that the synergistic work for prediction/simulation between factors in DATH may be more internally consistent than in DATC. The pH factor may represent a slightly higher relativity regarding the date–algae–temperature system, which can also indicate higher stability and anti-interference performance in applications. Therefore, in the real application test, the DATH model was selected to be evaluated with traditional simple process (SP) as a comparison.

### 3.2. The Prediction Performance in Real Applications

For real applications, as mentioned above, the performance of the DATH model was compared to the data acquired in 2022 (Table 4). Table 3 shows the statistical evaluation of the prediction performance for simulation in all conditions, with a simple process (SP) model as comparison. A drop in accuracy was found when compared to those data in the validation period of 2020 with the SP model, especially for the prediction of pH (Table 3 and Table 4). This could be due to the two-year gap between training and real-world application, where a change in background conditions over time may have occurred. However, the selected DATH system still shows an uncompromised prediction performance. This was attributed to a relatively closed system with high relativity between factors, where a good synergistic prediction could be conducted to achieve a higher anti-interference and more internally consistent performance.

**Figure 4:**
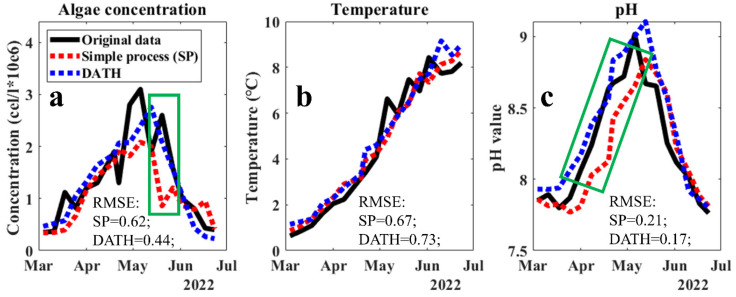
The prediction performance for (**a**) algae concentration, (**b**) temperature, and (**c**) pH value in the real application via DATH in 2022. The simple AI process (SP) was the control.

The prediction performance for algae, temperature, and pH in 2022 via the selected DATH model in application period was displayed in Figure 4. The performance of simple simulations with two factors (date and targeted factor) were for comparison. A satisfying performance for algae concentration prediction via DATH is documented in Figure 4a, even with a two-year gap between training and application periods. A significantly lower RMSE was obtained in DATH, compared to that in the simple simulation. A smooth prediction curve obtained through DATH helped to describe the important trends and slopes as well as to reveal general patterns. This can also be concluded for the pH prediction in Figure 4c. However, in Figure 4b, no significant gap between the two predictions of temperature was found. This may be due to (1) the higher degree of independence in temperature than in pH and algae concentration in the system, the application of closed systems for its prediction contributed limited help, and (2) its high regularity and periodicity leading to an easier prediction and diminished performance gap between different simulation methods. Therefore, the involvement of pH and algae concentration have little influence on the prediction performance of temperature. Overall, a higher-performance and relatively robust AI model for the prediction of algae concentration, temperature, and pH in the lake was obtained.

### 3.3. Discussion

The full scanning–focusing process resulted in two potential closed systems. The models of the DATH and DATC systems showed higher performances in the validation period compared to that of a blind process (BP) with all accessible data involved in training (Figure 2 and Figure 3). This indicates, on the one hand, that interventions of factors with low correlations between each other may lower the predictive performance of the FNN model. Additionally, on the other hand, in the application period (Figure 4), the higher performance of the DATH model compared to that of the simple process (SP) model suggested the importance of involving correlated indicators to improve the prediction accuracy and stability of performance. However, in terms of more independent factors, such as temperatures, which are not affected by water quality factors, the DATH model did not show significant differences in the SP model. The selection of highly correlated factors in our new system may contribute more to the prediction of dependent variables than independent variables. Overall, the closed system selected in this study showed the potential to further enhance the prediction of algae and other vital water quality factors in complex systems such as lakes.

Moreover, although in this work, the R^2^ of the prediction outputs by selected DATH and DATC models are not high, Figure 2, Figure 3 and Figure 4 showed the considerable matching of trends and changing of the curves between the predicted values and the measurements. In a complicated system of a natural lake environment with long term data, the controlling factors are beyond water quality, such as meteorological data. The accurate prediction of variables may not always be successful. In these cases, we believe that a good matching of trends and overall patterns significantly contributes to the purposes of prediction, especially when aiming for early warning systems where the trends and tipping points are more important than the actual numerical precision of the prediction. Early warning systems for the drinking water sector mainly provide indications of forthcoming changes and do not require precise predictions of the actual expected value. The risk of developing HABs in a lake only requires the knowledge of a previously agreed alarm level to put the necessary counteractive measures in place. Another benefit of the closed system approach presented here is the delivery of a model that predicts multiple factors. The indications of a forthcoming fast decrease in temperature or pH have other important consequences for water treatment. These predictions may serve to decide on other necessary important adjustments to be undertaken in the water treatment plant.

In addition, this study aims more to introduce a new system in order to improve prediction performance than pursue absolute high-accuracy prediction. This study provides another aspect for enhancing prediction quality. In recent years, other researchers have also outlined systems with good prediction performances for algae content in water. For instance, Saboe, S., et al. conducted LSTM modeling to predict blue green algae (BGA), chlorophyll, conductivity, etc., with designed parameters and indicators as inputs [13]. We believe that a full-scanning–focusing process may contribute to further improving prediction by selecting training parameters and input factors. Silva, A., et al. used a support vector machine (SVM) model to predict future dinophysis acuminata cells and considered three groups of indicators: (1) past cells abundance, (2) environmental data. and (3) combined prediction, resulting in fair accuracy [21] This, on one hand, suggested the advantages of indicating factor selection and, on the other hand, showed the potential for further improving the prediction in the future by selecting specific highly correlated closed systems, as we introduced in this study.

Our study aims to explore a computational method of using optical sensors for improving water quality predictions. Our study, on one hand, benefits practical routine work at water utilities by saving money and time, compared to traditional manual work/counting in the laboratory. On the other hand, optical sensors have many advantages compared to remote sensing methods, such as those discussed by Rocher and colleagues: namely that the optical sensors are not affected by clouds and are flexible in their application in different parts of a water system [22].

This study also has limitations. Firstly, the numerical prediction model obtained in this study can be a good basis for developing new early warning systems. However, the establishment of an early warning system still requires more effort to combine the prediction and judgmental models/algorithms. This will be our next development step in the future. Secondly, the accuracy of prediction can be further improved by considering more AI models, data pre-treatment methods, etc. Moreover, more training parameters and potential water quality indicators/factors should be included in the future to further enhance prediction performances.

## 4. Conclusions

The variations in algae concentration and other vital factors in the Görväln drinking water plant were simulated and predicted using our new AI scanning–focusing process. In the relative closed systems (1) date–algae–temperature–pH (DATH) and (2) date–algae–temperature–CO_2_ (DATC) it was found that the factors therein had high relativities in time-series compared to those with other factors. The predictions using those systems showed dominating advantages compared to those of the blind prediction (BP) model with all accessible factors during training. DATH displayed more stable predictions compared to that of DATC, this may be due to the vital effect of carbon dioxide concentration through factors outside the DATC. In the real application test, DATH showed an outstanding prediction performance, despite the monitored data obtained two years after those of the training period. The prediction showed higher accuracy when describing the values and trends compared to the simple process (SP), which only involved the date and algae concentration. In general, the higher performance of the new models was derived from the scanning–focusing process, which selected both the best model and highest relative closed system. Our AI scanning–focusing process and model selection showed the potential for improving water quality prediction by identifying closed systems with highly correlated factors. This provides a new method that can be considered in the enhancement of numerical prediction for factors in water quality monitoring and wider environmental applications.

The study still has limitations in terms of further improving prediction performance, the need to involve more parameters and water quality indicators, and the establishment of an entire early warning system. This leaves room for future study.

In the next steps, we plan to further improve this modeling process by involving more parameters of modeling into the scanning and selection procedures and producing the best models that can be realized within the framework of data measured in concert and NN modeling. For the early warning system purposes, our process will be combined with AIs for recognition and classification to achieve high performances for predicting risky turns and state-changing points. The target of development is to build a high-performance AI process for prediction and early warning systems not only for the evaluation of water quality but also for other research and industrial areas.

## Figures and Tables

**Table 1:** List of sensors installed at the raw intake point that are relevant for this study, including pH, temperature, conductivity, turbidity, manufacturer, approximate precision, data acquisition rate, and the median and range of observed values during the study period of 1 January 2016–31 December 2021.

Sensor Type	Instrument Manufacturer	Precision	Data Acquisition Interval (mins)	Observed Range
pH	Knick	0.05	5	7.2–9.3
Temperature		0.01 [°C]	5	0–12.5
Conductivity	Yokogawa	1 [mS/m]	5	20–35
Turbidity	Hach Aquatrend	0.005 [FTU]	5	0–30

**Table 2:** Statistical evaluation of performance during the training period (2015–2019). BP refers to a blind AI training process with all factors considered as inputs, and SP refers to a simple process with only time and target factors as inputs.

Evaluation of Fitting Performance in the Training Period								
Methods	Algae Conc.		Temperature		pH		CO_2_ Conc.	
	R^2^	RMSE	R^2^	RMSE	R^2^	RMSE	R^2^	RMSE
SP	0.613	0.307	0.912	1.071	0.856	0116	0.988	2.25 × 10^−6^
BP	0.836	0.199	0.948	0.82	0.894	0.1	0.979	3.00 × 10^− 6^
DATH	0.64	0.296	0.903	1.125	0.872	0.109		
DATC	0.631	0.299	0.906	1.107			0.988	2.30 × 10^−6^

**Table 3:** Statistical evaluation of performance in the validation period (2020). BP refers to a blind AI training process with all factors considered as inputs and SP refers to a simple process with only the time and target factors as inputs.

Evaluation of Fitting Performance in Validation Period								
Methods	Algae Conc.		Temperature		pH		CO_2_ Conc.	
	R^2^	RMSE	R^2^	RMSE	R^2^	RMSE	R^2^	RMSE
SP	0.461	0.868	0.97	0.412	0.823	0.184	0.977	2.27× 10^−6^
BP	0.081	1.133	0.422	1.821	0.361	0.35	0.702	8.71 × 10^−6^
DATH	0.243	1.028	0.886	0.808	0.844	0.173		
DATC	0.398	0.917	0.809	1.047			0.788	7.35 × 10^−6^

**Table 4:** Statistical evaluation of the performance during the application period (2022). SP refers to a simple process with only the date and target factors as inputs.

Evaluation of Fitting Performance in the Application Period						
Methods	Algae Conc.		Temperature		pH	
	R^2^	RMSE	R^2^	RMSE	R^2^	RMSE
SP	0.452	0.619	0.944	0.668	0.703	0.214
DATH	0.719	0.443	0.932	0.732	0.808	0.173

## Data Availability

Restrictions apply to the availability of these data. Data was obtained from Norrvatten AB and are available from linda.holmer@norrvatten.se with the permission of Norrvatten AB.
